# Media sclerosis drives and localizes atherosclerosis in peripheral arteries

**DOI:** 10.1371/journal.pone.0205599

**Published:** 2018-10-26

**Authors:** Pak-Wing Fok, Peter Lanzer

**Affiliations:** 1 Department of Mathematical Sciences, University of Delaware, Newark, Delaware, United States of America; 2 Mitteldeutsches Herzzentrum, Standort Klinikum Bitterfeld, Bitterfeld, Germany; Centro Cardiologico Monzino, ITALY

## Abstract

Media sclerosis (MS) and peripheral artery disease (PAD) may coincide, particularly in type 2 diabetics (T2D) and in patients with chronic kidney disease (CKD). In contrast to non-diabetics, in T2D PAD is more severe and more distal. Although MS is suspected to play a role, the underlying pathophysiological reasons for the differences still remain elusive today. We tested the hypothesis that MS is a promoter of atherosclerosis as it occurs in T2D with PAD by interfering with arterial remodeling using an *in-silico* simulation. We confirmed that MS aggravates PAD by promoting negative remodeling. We found that the effect is more pronounced in smaller distal arteries compared to larger proximal ones. Our results suggest that the degree of this divergence depends on the ratio between the thickness of the intima relative to the thickness of the media/adventitia of the individually affected arteries.

## Introduction

In type 2 diabetics (T2D) and in patients with chronic kidney disease (CKD) peripheral arterial disease (PAD) is common [1–3] and frequently coincides with media sclerosis (MS) [4, 5] Compared with non-diabetics, in T2D PAD is more severe and more distal [6].

**Fig 1:**
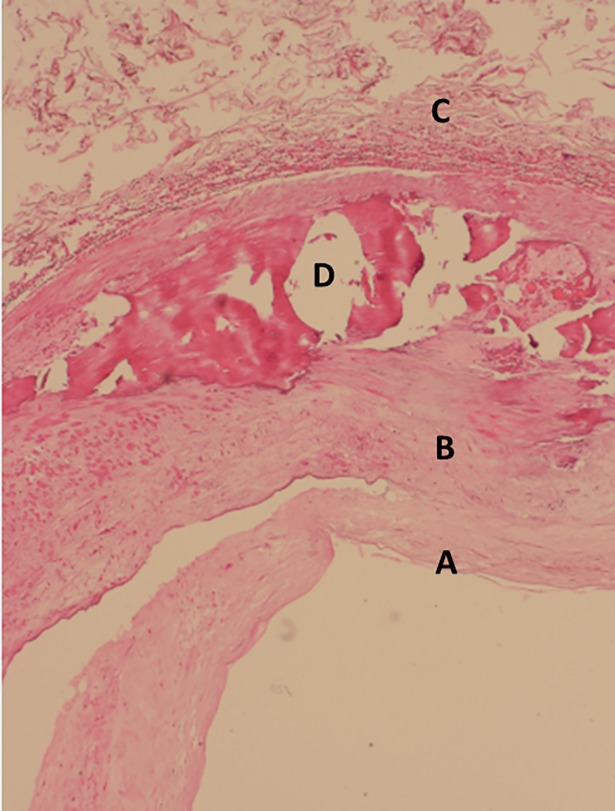
Histological section of a popliteal artery with advanced MS in a 65 years old male T2D. In a hematoxylin- eosin stained histogram calcified plate (D) in the media (B) can be seen; A – intima, C - adventitia.

While atherosclerosis is a disease of the intima, characterized by progressive plaque formation [7], MS is a disease of the media characterized by progressive calcifications [8]. Fig 1 shows a histological section of a popliteal artery from a patient with MS showing massive calcifications within the media.

Since MS primarily does not affect the intima, historically, it was thought of as an “innocent bystander” to peripheral arterial atherosclerosis [9]. However, more recently MS has been recognized as a major independent cardiovascular risk factor and a powerful negative prognostic indicator [10]. One startling consequence of MS is its effect on blood flow. Efficient circulation of the blood relies on the elasticity of vessel walls and transmission of pressure gradients acting in concert to create “vis a tergo” forces that pump the blood throughout the body. Vessels with advanced MS lose their compliance and their ability to stretch and recoil; consequently, “vis a tergo” is gradually lost [11]. In advanced cases there can be a virtual shutdown of peripheral blood flow.

## Hypothesis

How blood vessels change their morphology due to the progression of atherosclerosis is a fundamental question in cardiovascular medicine. In 1987, Glagov et al. [12] provided a partial answer to this question by examining coronary arteries. They proposed that the lumen area of arteries remains roughly the same size (positive remodeling) before decreasing (negative remodeling), as atherosclerosis progresses. Thus, Glagov et al. hypothesized that vessel remodeling occurs in two phases: a compensation phase followed by a stenotic phase.

Based on Glagov’s findings, a given increase in intimal area can result in maintenance or a decrease in normalized lumen area (NLA). However, Glagov’s analysis says very little about remodeling rates and how vessel geometry affects these rates. For example the mechanisms responsible for the greater severity and the distal location of PAD in T2D are not clearly understood. We hypothesized that a) MS forcibly promotes atherosclerosis as clinically seen in T2D with PAD and in patients with chronic renal disease by interfering with the remodeling process and b) the severity of interaction depends on vessel sizes; the smaller the vessel the more severe the interference. In the absence of a suitable experimental model of MS we tested this hypothesis using a mathematical model and computer simulations.

### Hypothesis testing *in-silico*

In the absence of longitudinal studies of T2D with PAD and MS, and in the absence of suitable experimental animal models of diabetic PAD and/or MS, we were unable to test our hypothesis in *in-vivo* settings. Therefore, we developed an *in- silico* model (see S1 Appendix for details) allowing the testing of our hypotheses using computer simulation. To ascertain realistic scenarios of the human disease, the simulation was based on the limited clinical data available in the literature.

The luminal diameter and the arterial wall thickness of above-the-knee (ATK) arteries (superficial femoral artery) and below-the-knee arteries (tibial anterior, tibial posterior and peroneal) varies in normal subjects depending on a number of factors such as age, sex and level of exercise but also on the method of measurement; on average luminal diameters of approximately 7mm and 3mm for ATK and BTK arteries, and arterial wall thicknesses of about 0.8 and 0.3mm, respectively, appear to be realistic [13–18]. While in healthy subjects, the intima initially consists of a single layer of endothelial cells supported by an elastic lamina, in subjects with PAD the thickness markedly increases as atherosclerosis progresses [19].

**Fig 2:**
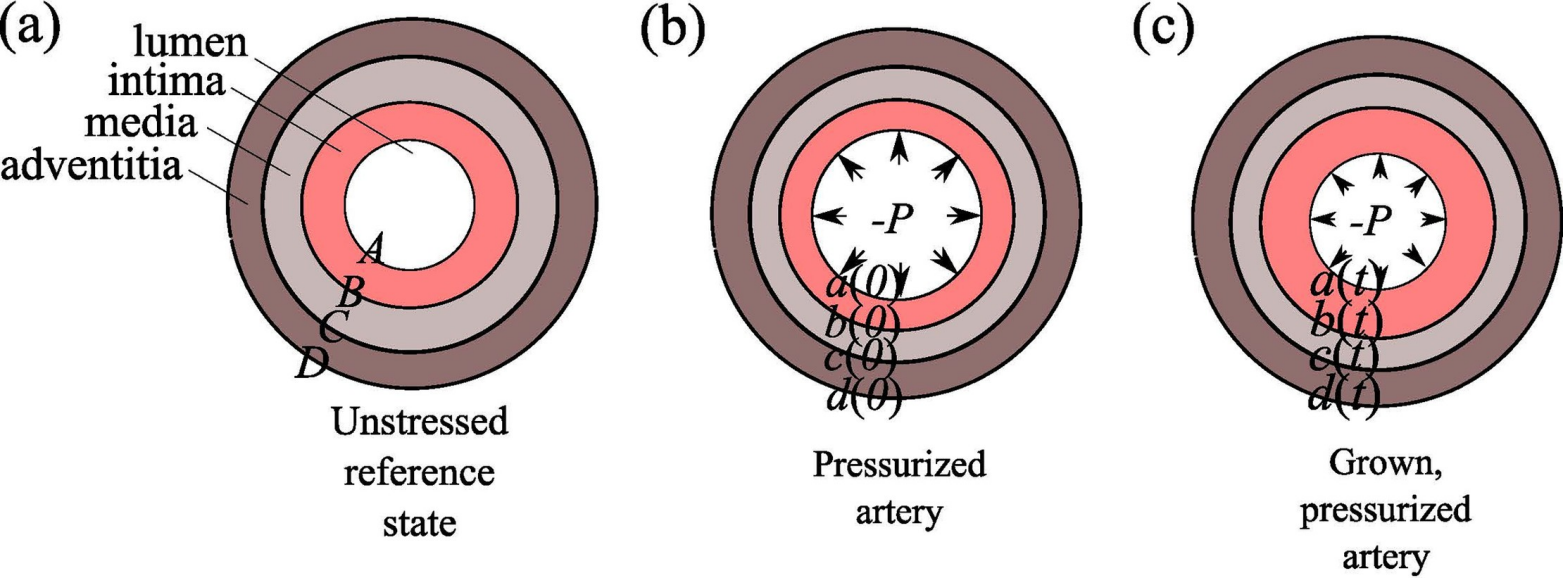
Three layers model of the arterial wall. (a) The unstressed reference state of a multi-layer annulus defined by *A<R<D*. (b) A pressurized artery with no intimal growth at time *t=0*. The intima is healthy and its thickness, usually on the order of 5-10 μm, is exaggerated for clarity. (c) A grown, pressurized artery at time *t>0*; the annulus is now defined by *a<r<d*. The lumen pressure is *P* and the intima is significantly thicker than in (b). The radial coordinate in the reference frame is denoted R and it ranges from A (radius of lumen) to D (radius of the boundary of the adventitia). The lower case letters denote positions in the deformed frame; thus r is the radial coordinate in the deformed frame and it ranges from a (radius of deformed lumen) to d (radius of the boundary of the deformed adventitia).

To represent the morphology of the human leg arteries, our mathematical model [20] describes the growth and elastic deformations of an idealized arterial cross section with three concentric layers: the intima, media and adventitia (Fig 2).

Each layer has its own thickness and stiffness to mimic varying degrees of MS. PAD is simulated by allowing the intima to grow. This framework allows us to study and understand the interactions between MS and PAD, providing a way to test our hypothesis *in-silico*. Note that our model does not explicitly address T2D. It only describes vessel walls in the absence or presence of media sclerosis and atherosclerosis. However, as T2D patients are more likely to have MS we assumed that MS vessels represent a convenient model for T2D vessels.

## Results

**Fig 3:**
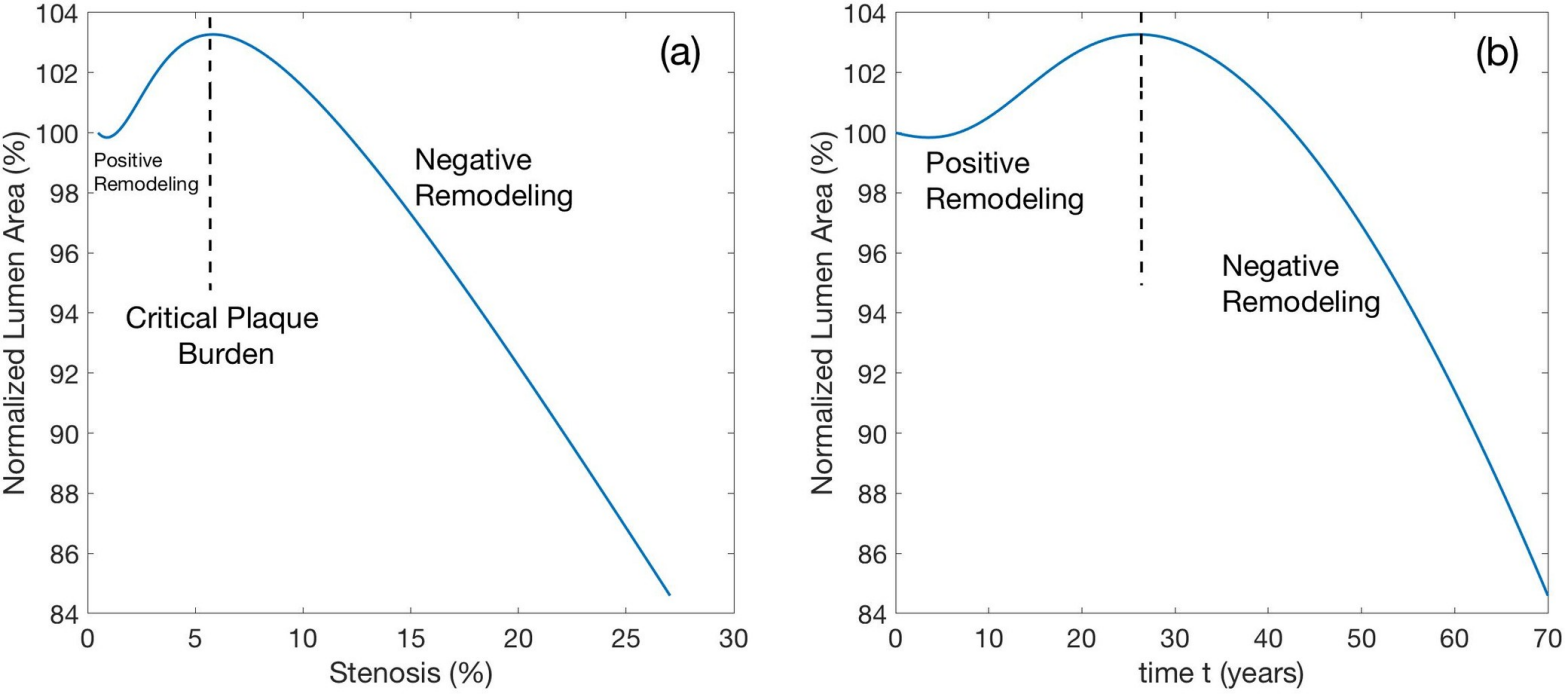
Remodeling of a femoral artery with atherosclerosis in the absence of MS. Media and adventitia properties are static in time. a) The artery remodels in two stages with the transition occurring at a critical plaque burden of about 6% occurring at approximately 28 years of age. Before the critical state, positive remodeling occurs, and the lumen area slightly increases. After the critical disease state, the lumen area is steadily decreasing. b) The cumulative effect of aging on the normalized lumen area (NLA). The behavior is qualitatively similar to a).

First, we modeled the effect of pure atherosclerosis on stenosis behavior (Fig 3A and 3B). In the context of our model, stenosis is defined as

$$\mathrm{Stenosis}=\frac{\mathrm{Intima}\;\mathrm{Area}}{\mathrm{Lumen}\;\mathrm{Area}+\mathrm{Intima}\;\mathrm{Area}}=1-\frac{a^{2}(t)}{b^{2}(t)},$$

where *a* and *b* are radii of the lumen and internal elastic lamina (Fig 3C). The Normalized Lumen Area (NLA) is defined as

$$NLA(t)=\frac{a^{2}(t)}{a^{2}(0)}.$$

Consistent with Glagov’s results [12], we see in Fig 3A that for mild stenosis (up to about 6% in this case), the artery is able to compensate for an increase in intimal mass and during this stage the lumen area *increases* for increasing plaque burden (positive remodeling). Beyond a critical plaque burden, there is a persistent decrease in lumen area as atherosclerosis progresses (negative remodeling); a possible mechanical explanation for this phenomenon has been discussed previously [20]. In Fig 3B it can be appreciated that the same effect occurs when NLA is evaluated as a function of time. According to the simulation results, given a steady and continuous progression of atherosclerosis throughout the lifetime of a person, the critical plaque burden occurs when the hypothetical patient is at the critical age of about 28 years. Interestingly, although our calculations show that the cross-sectional area of the intima at 70 years of age becomes 64x larger compared to the outset, the lumen area has reduced only by about 16%, illustrating the remarkable capability of large non-MS arteries to maintain their lumina despite the virtually lifelong progression of atherosclerotic plaques.

**Fig 4:**
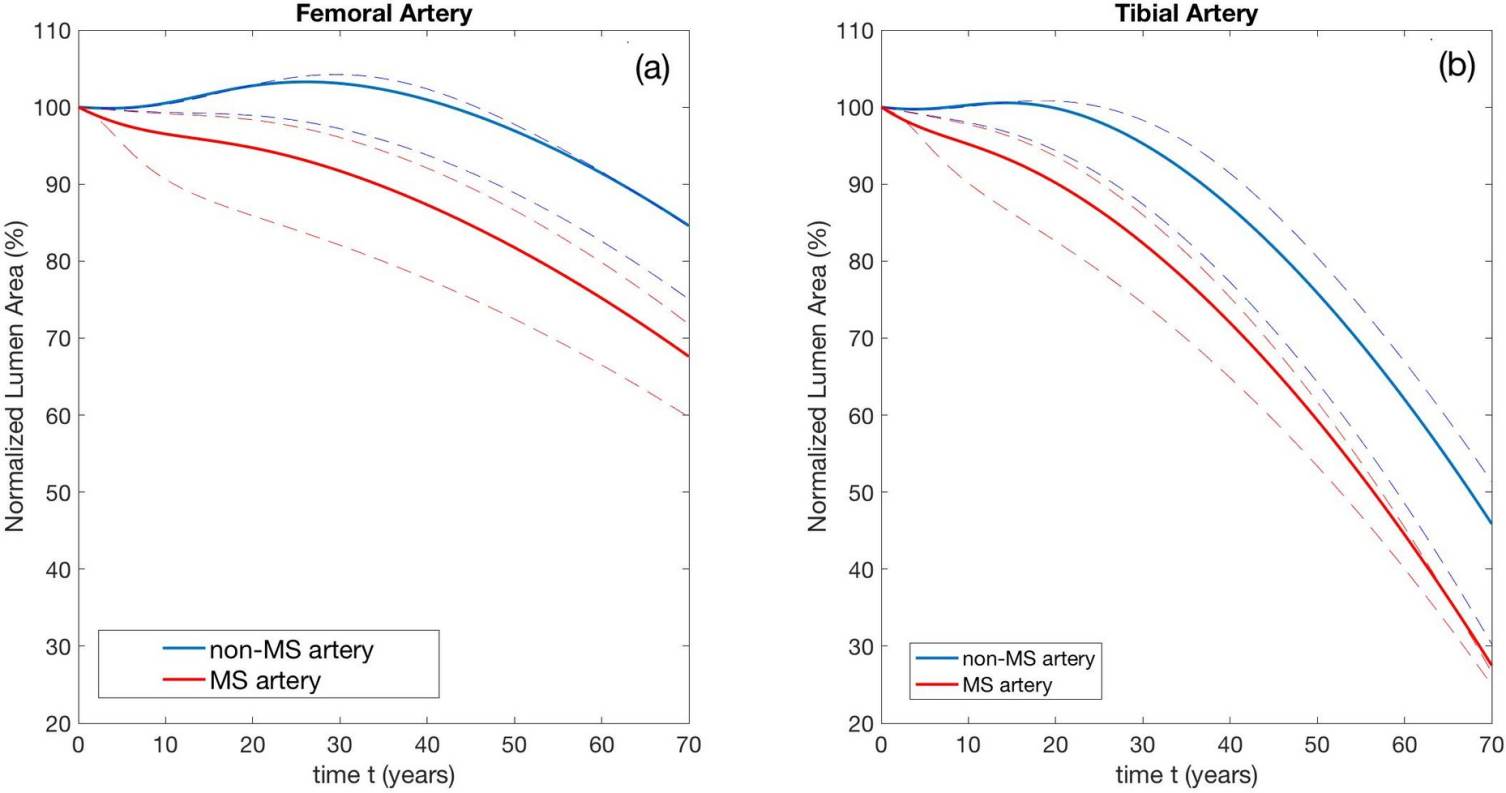
**Remodeling curves for (a) femoral and (b) tibial arteries in the absence (blue) and presence (red) of MS throughout of a hypothetical life span (70 years) of an individual person.** a) In femoral arteries the reduction of the Normalized Lumen Area (NLA) in patients with atherosclerosis without MS is approximately 15% and about 30% in patients with atherosclerosis and MS. b) In tibial arteries the reduction of the NLA in patients with atherosclerosis and without MS is approximately 55% and about 75% in patients with atherosclerosis and MS. Intima and media parameters and associated stiffening rates are given in Tables B, D, E, and F in S1 Appendix. Non-MS media stiffening rates are 1/10 of MS stiffening rates. Dashed lines represent lower and upper bounds, estimated by taking the largest and smallest (non-zero) values of $c_{0}^{*},\;c_{1}^{(2)*},c_{2}^{(2)*},c_{1}^{(3,4)*}$ and $c_{2}^{(3,4)*}$ from reference [21].

Next, we modeled the effect of MS on developing atherosclerosis. Fig 4 shows remodeling curves for non-MS and MS femoral and tibial arteries, respectively.

In Fig 4A we see that femoral arteries with PAD but no MS maintain their NLA at or above 100% for about 30 years before experiencing a progressive but modest decrease in the final 40 years. In addition, there is initially a definite and distinctive phase of positive remodeling where NLA increases. In contrast, the tibial arteries (Fig 4B), start to remodel inwardly much earlier, at 20 years of age, and the lumen loss is much more significant. By the end of the patient’s life, NLA is less than 50%, illustrating the greater propensity of smaller BTK arteries to develop stenoses. Furthermore, in tibial arteries the phase of positive remodeling is virtually absent.

When PAD and MS are concurrent, the remodeling curves for both the larger femoral and the smaller tibial arteries are modified as shown by the red curves in Figs 4A and 4B. Clearly, the early phase of positive remodeling is completely lost; in both types of arteries with PAD and MS, negative remodeling always occurs. Remarkably, our simulations show that femoral and tibial arteries that are afflicted by PAD *and* MS present NLAs that are consistently smaller than NLAs of arteries with PAD alone. This difference is maintained throughout the lifetime of the patient. At the end of the patient’s life in the larger femoral arteries, the presence of concurrent MS accounts for at least an additional 15% of luminal loss, bringing the total NLA loss to about 30%. In contrast, for the smaller tibial arteries, while the luminal loss due to the concurrent presence of MS is similar (about 15%), at the end of patient’s life the total loss of lumen is far greater, at about 70%.

**Fig 5:**
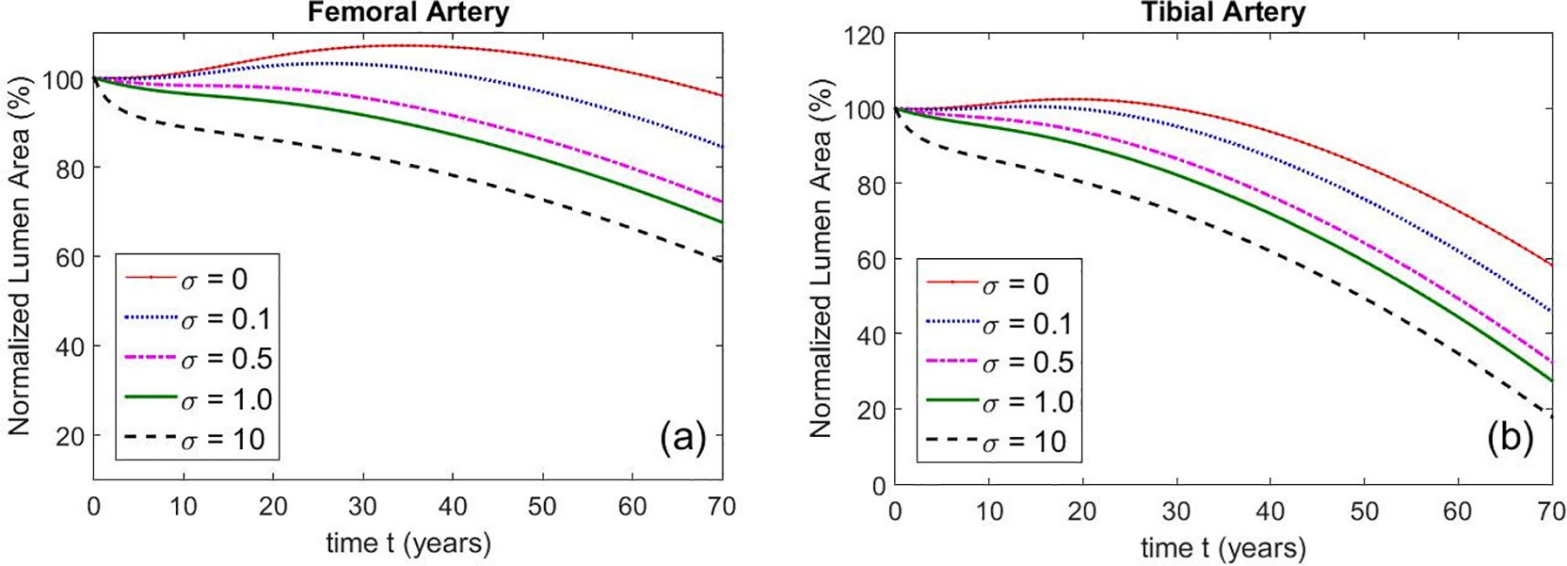
**Femoral (a) and tibial (b) remodeling curves for different media stiffening rates.** The stiffening rates are multiplied by the parameter σ A hypothetical media whose stiffness remains static in time is represented by σ = 0, while larger values of σ represent a more aggressive MS development (σ=1 corresponds to the values of *λ*_0_, $\lambda_{1}^{\left(2\right)},\;\lambda_{2}^{\left(2\right)},\lambda_{2}^{\left(2\right)}\;\mathrm{and}\;\lambda_{1}^{\left(3,4\right)}$ in Table E in S1 Appendix).

Importantly, as shown in Fig 5, we obtain qualitatively similar results when we change the stiffening rate in the media. Specifically, we take the media stiffening rates to be *σλ*_0_, ${\sigma \lambda }_{1}^{\left(2\right)},{\sigma \lambda }_{2}^{\left(2\right)},{\sigma \lambda }_{2}^{\left(2\right)}\;\mathrm{and}\;{\sigma \lambda }_{1}^{\left(3,4\right)}$ where σ is an adjustable parameter and *λ*_0_, $\lambda_{1}^{\left(2\right)},\;\lambda_{2}^{\left(2\right)},\lambda_{2}^{\left(2\right)}\;\mathrm{and}\;\lambda_{1}^{\left(3,4\right)}$ are base rates parameters estimated from [21] (Table E in S1 Appendix).

By taking σ larger, we can simulate more aggressive MS development, with σ = 0 corresponding to a hypothetical media whose material properties remain static in time. We see that for a given value of σ tibial arteries still experience more inward remodeling than femoral arteries, resulting in smaller NLA. As expected, larger values of σ result in smaller lumen areas.

The intima of healthy arteries typically consists of a single layer of endothelial cells and a basement membrane with a total thickness of 5 – 10 *μ*m. This thin layer initially lines all arteries no matter how large or small they are. In contrast, the thickness of the media and adventitia is highly variable depending largely on the size and type of artery. The greater susceptibility of BTK arteries (compared to ATK) to negative remodeling appears to be related to the differences in their intima-to-media thickness ratios that typically *increase* with *decreasing* vessel size.

**Fig 6:**
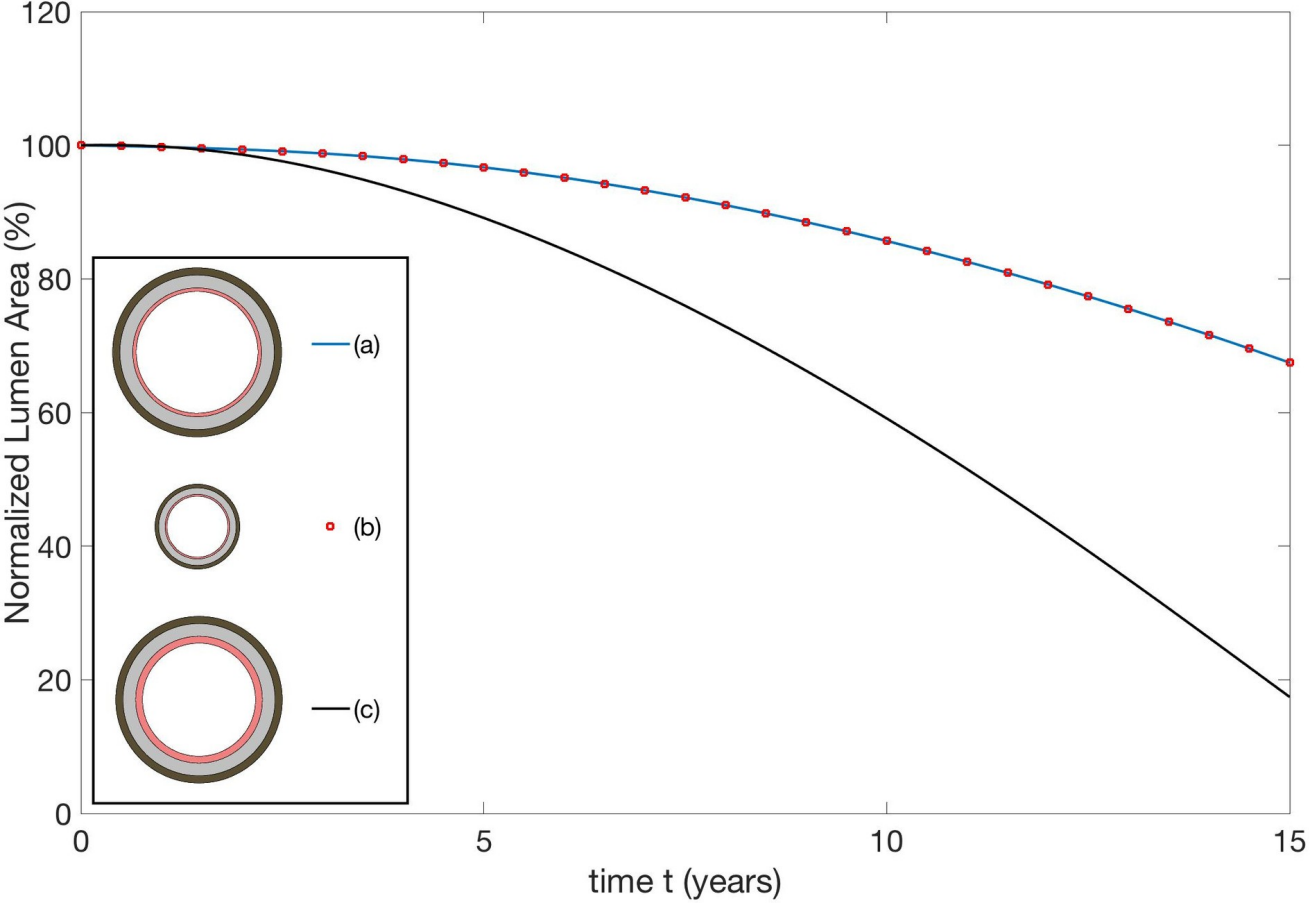
Remodeling curves for three arterial cross sections. The cross sections (a) and (b) are geometrically similar with the dimensions of (b) half that of (a). The cross section (c) has identical media and adventitia thickness to (a), but the initial intima thickness is doubled. See text for exact dimensions.

Fig 6 illustrates this finding.

The red-dotted remodeling curve (a) corresponds to a vessel with reference intima thickness 0.2 mm and cross-sectional dimensions *A* = 3.5 mm, *B* = 3.7 mm, *C* = 4.44 mm, *D* = 4.86 mm. The blue remodeling curve (b) corresponds to a geometrically similar vessel with reference intima thickness 0.1 mm and *A* = 1.75 mm, *B* = 1.85 mm, *C* = 2.22 mm and *D* = 2.43 mm: all dimensions are reduced by 50%. The remodeling curves are identical and NLA reduces from 100% (at birth) to about 70% after 15 years for both arteries. This invariance principle is simply a restatement of the fact that the curve does not depend on the units used to measure *A*, *B*, *C* and *D*. The black curve (c) corresponds to a cross section with the same media and adventitia wall thicknesses as (a) but the initial intima thickness has been doubled so that *A* = 3.3 mm, *B* = 3.7 mm, *C* = 4.44 mm and *D* = 4.86 mm. We see that aggressive inward remodeling occurs and the NLA shrinks much faster than before, reducing from 100% to 20% in just 15 years. These remodeling curves are for non-MS arteries with parameters given by Tables A-F in S1 Appendix and the stiffening rates *λ*_0_, $\lambda_{1}^{\left(2\right)},\;\lambda_{2}^{\left(2\right)},\lambda_{2}^{\left(2\right)}\;\mathrm{and}\;\lambda_{1}^{\left(3,4\right)}$ are 1/10 of those reported in Table E in S1 Appendix.

These results show that it is the relative thicknesses of the intima, media and adventitia that determine the lumen evolution. The primary determinant of remodeling behavior is not intima thickness *per se*, but its thickness in relation to the lumen radius, media thickness and adventitia thickness. Thicker intima tends to remodel inwardly because it is more energetically favorable from a mechanics perspective. All other things being equal, to dilate the lumen of a vessel by a given percentage requires more energy if the intima is thicker.

## Discussion

MS and atherosclerosis, manifested as PAD, frequently coincide in T2D and in patients with CKD [3– 5] Compared to non-diabetics, the PAD in T2D is more severe [1, 22] and preferentially affects smaller below-the-knee arteries [6, 23]. To date this anomaly has not been adequately explained. Although data on vascular morphology obtained from leg amputation specimen have been published [24, 25] to our knowledge data on mechanical properties of the individual layers of arterial walls in patients with MS are not available. Using mathematical modeling and computer simulation we were able to show that this clinically relevant inconsistency can be explained by media sclerosis interfering with arterial remodeling. In addition, we were able to show that the degree of this interference depends on the arterial wall geometry (Fig 4).

### The impact of media sclerosis on atherosclerosis

While MS and atherosclerosis frequently coincide, as seen in T2D with PAD, their interactions have not yet been explored. By modelling both diseases we were able to show that if atherosclerosis and MS coincide, MS enhances the stenotic process associated with atherosclerosis by persistently promoting inward remodeling. More specifically, arteries with atherosclerosis and MS consistently present lumina that are smaller than arteries with atherosclerosis alone. The degree of reduction depends on the stage of atherosclerosis but can be up to 15% towards the end of the patient’s lifetime. Intuitively, gradual stiffening of the media associated with MS interferes with positive Glagov remodeling (GR) in early stages of atherosclerosis while in more advanced stages; MS promotes negative GR by preventing the media from undergoing adaptive expansion.

### The role of intima – media thickness ratios on peripheral artery atherosclerosis

Our results show that because arteries in the body are generally geometrically dissimilar (i.e. they are not scaled versions of each other), there is no universal pattern of GR. Above-the-knee (ATK) and below-the-knee (BTK) arteries follow different patterns of GR depending on the relative sizes of all three arterial wall layers. If atherosclerosis alone is present, smaller BTK arteries are more adversely affected than larger ATK arteries. Thus, after the same amount of time and the same burden of atherosclerotic plaque buildup, smaller BTK arteries have lumina that are smaller than predicted, even after accounting for initial geometric differences in size. Stated differently, intimal thickening associated with atherosclerosis drives different patterns of GR depending on the relative dimensions of the arterial wall layers in individual arteries. This divergent effect is further accentuated in the presence of MS.

### Medical implications

PAD in T2D is detrimental to quality of life and increases their risk of disability and fatal and non-fatal cardiovascular events, including limb loss, compared to non-diabetics [1, 26–28]. Compared to non-diabetics, PAD in T2D is more severe, more diffuse, and located mainly in BTK arteries [6, 23]. Our modeling effort supports and explains these clinical observations. Furthermore, it stresses the importance of MS in T2D and CKD patients with PAD in cardiovascular prevention. For example, early detection of MS could justify early anti-atherosclerotic measures including vigorous prevention, close monitoring of disease progression and revascularization measures.

Although we presented our results over a time scale of 70 years, our model is able to describe MS progression over shorter or longer periods of time, and at different rates, with simple modifications. Thus, using our model it appears feasible to study MS as it occurs in humans. Given the absence of suitable animal models, the study of MS using computer modelling could have a number of clinically relevant applications. For example, the impact of antidiabetic therapy on the progression and interaction of PAD and MS could be assessed. Thus, while we predict that MS promotes differential remodeling, further tests must be done to validate our model. Furthermore, besides geometric factors, other causes such as differences in hemodynamics may be also important [29–30]. More recently, the effects of exercise on gene expression have been reported [31]. Furthermore, heterogeneity of molecular genetics among endothelial cells [32] and vascular smooth muscle cells [33] across different vascular beds may also play an important role in expression of vascular diseases including propensity to calcifications [34]. Although, the genetic predisposition is also likely to modify the vessels sizes and possibly even the arterial wall layering, to our knowledge no data addressing this important question are available in the literature.

## Limitations and assumptions

An important limitation of the model is that the predicted behavior of the arteries depends critically on the material properties and parameters used in the strain energy function. Although these parameters for individual layers in femoral and tibial arteries are not available, Kamenskiy et al. [21] performed mechanical tests on femoral, popliteal and tibial arteries and published the parameters for the vessel wall as a whole. We used these values to guide our estimates, assuming the properties of the media and adventitia are identical initially. As atherosclerosis progresses, we simulate a calcified media by increasing its material parameters linearly in time while assuming that the properties of the adventitia remain static. The selected rate of increase was estimated from the Kamenskiy et al. data set. The limitation here is that there may be relevant inter- and intra-patient variability in the vessel wall properties depending on environment, lifestyle and genetic makeup. Our results do not necessarily give the “average” remodeling behavior within a population; rather they give remodeling curves for idealized *ex-vivo* arteries with the material properties specified in Tables A-F in S1 Appendix.

To calculate the stiffening rates, we selected the data from two patients, number 5 and 10 from Kamenskiy et al. for the following reason. Patient 5 had the least advanced atherosclerosis (labeled as “nearly normal tissue”) while patient 10 had the most advanced atherosclerosis (labeled as “severely diseased artery”); both patients had no other disease documented as a possible cause for the amputation. Because data on a single patient over time cannot be obtained due to amputation, to quantify the progression of calcification, patients with early and late stages of atherosclerotic disease had to be selected, with a compliant and stiff media respectively. Using the data from other patients (e.g. patients 7 and 8) would have resulted in negative stiffening rates (i.e. media that becomes less stiff over time).

Our model also neglects feedback and mechano-sensing of the artery. Studies from the 1980s [35, 36] have shown that vessel morphology can adapt to changes in shear stress through the activation and release of vasodilators or vasoconstrictors from the endothelium. Such effects are not included in our model. We also used a model for tissue growth that assumes that the artery is perfectly elastic. In practice, tissues in the vessel wall can exhibit viscoelastic behavior and hysteresis, and both of these effects were neglected.

We also address the geometric assumptions underlying our model. MS arteries appear to have media that is slightly thicker than non-MS arteries. In addition, in advanced cases, patchy thickening due to nodular bone-like formations may occur [8]. Our model neglects this modest effect and it is not likely to significantly alter our results. It is also important to note that atherosclerosis in patients with PAD progresses due to repeated plaque rupture. Combined with blood clot formation and consecutive integration into the vessel wall, the process occurs in a rather discontinuous and patchy manner while, at least initially, MS progresses in a more steady and gradual fashion. In our model, both PAD and MS were modeled as gradual, time-continuous processes.

Finally, we note that our mathematical model posits the existence of a reference configuration (see Fig 2(A)), from which we mathematically compute grown and deformed states (see Fig 2B and 2C). Growth is always defined with respect to the reference configuration. Thus, diseased states with time-varying intima/media properties are simulated by first changing the material properties in the reference configuration and then allowing the intima to grow to its target value. The reference state corresponds to an unstressed vessel with no disease or lumen pressure. However, real blood vessels and real intima layers are always residually stressed, even when no lumen pressure present.

## Supporting information

S1 Appendix Details and parameters of mathematical model. (DOCX)
