# Supplementary material for: Media sclerosis drives and localizes atherosclerosis in peripheral arteries
Source: PLoS One. 2018 Oct 26;13(10):e0205599. doi: 10.1371/journal.pone.0205599 (PMC6203409; doi:10.1371/journal.pone.0205599)
Supplement: S1 Appendix — (DOCX) [file pone.0205599.s001.docx]

**Supporting information (S1 Appendix S1 Appendix. Details and Parameters of Methematical Model)**

**)**

**Morphoelasticity model of arterial cross section**

When arteries are pressurized, they take on an approximately circular cross-section. We therefore developed a morphoelastic model of a multi-layer artery where the intima, media and adventitia are described as concentric circular layers. Our goal is to investigate the deformation of the cross-section under the joint action of intimal growth and a calcifying media. The model closely resembles the one discussed in [20]: see Fig 2.

Since the development of PAD occurs slowly compared to the relaxation of any induced stresses in the artery, at every stage of the growth we solve for the elastic deformation of the vessel layers by assuming mechanical equilibrium. Growth is accounted for by decomposing the deformation gradient $F$ into a product of the elastic tensor $F_{e}$ and a growth tensor $F_{g}$*:*

$$F=F_{e}F_{g}.$$

Aiming for the simplest model that can describe the effects of MS, growth is assumed to be isotropic in the radial and azimuthal directions so that $F_{g}=diag(g,g,1)$ where *g(t)* is a prescribed function: material area elements in the grown state Fig 2(c) are $g^{2}$times larger than in the reference state Fig 2(a). We assume that deformations are independent of azimuthal angle or axial distance along the artery. The outer boundary of the adventitia wall is assumed to be traction-free while the inner boundary is held at a given lumen pressure of 120 mmHg. Blood flow and the effect of wall shear stress are neglected. In the context of atherosclerosis, these effects are thought to affect disease progression which is already accounted for in our model through the parameter *g*. Dimensions of the arterial sections in their reference configuration are given in S1 Table A.

The mechanical properties of the intima, media and adventitia are defined by hyperelastic strain energy functions. Following Kamenskiy [21]*,* the strain energy functions *W*_1_, *W*_2_ and *W*_3_ for the intima, media and adventitia are assumed to take the form:

$$W_{1}=\frac{b_{0}}{2}\left( I_{1}-3 \right)+\frac{b_{1}^{\left( 2 \right)}}{4 b_{2}^{\left( 2 \right)}}\left\{ \left. \exp\left[ b_{2}^{\left( 2 \right)}\left( I_{4}^{\left( 2 \right)}-1 \right)^{2} \right]-1 \right\} \right.+\frac{b_{1}^{\left( 3,4 \right)}}{2 b_{2}^{\left( 3,4 \right)}}\left\{ \left. exp[b_{2}^{\left( 3,4 \right)}\left( I_{4}^{(3,4)}-1 \right)^{2}]-1 \right\} \right.,$$

$$I_{1}=\lambda_{r}^{2}+\lambda_{\theta}^{2}+\lambda_{z}^{2},$$

$$I_{4}^{(2)}=\lambda_{\theta},$$

$$I_{4}^{(3,4)}=\cos^{2} \beta+\lambda_{\theta}\sin^{2} \beta,$$

$$W_{2}=\frac{c_{0}}{2}\left( I_{1}-3 \right)+\frac{c_{1}^{(2)}}{4 c_{2}^{(2)}}\left\{ \left. exp[c_{2}^{\left( 2 \right)}\left( I_{4}^{(2)}-1 \right)^{2}]-1 \right\} \right.+\frac{c_{1}^{(3,4)}}{2 c_{2}^{(3,4)}}\left\{ \left. exp[c_{2}^{\left( 3,4 \right)}\left( I_{4}^{(3,4)}-1 \right)^{2}]-1 \right\} \right.,$$

$$I_{4}^{(3,4)}=\cos^{2} \gamma+\lambda_{\theta}\sin^{2} \gamma,$$

$$W_{3}=\frac{d_{0}}{2}\left( I_{1}-3 \right)+\frac{d_{1}^{(2)}}{4 d_{2}^{(2)}}\left\{ \left. exp[d_{2}^{\left( 2 \right)}\left( I_{4}^{(2)}-1 \right)^{2}]-1 \right\} \right.+\frac{d_{1}^{(3,4)}}{2 d_{2}^{(3,4)}}\left\{ \left. exp[d_{2}^{\left( 3,4 \right)}\left( I_{4}^{(3,4)}-1 \right)^{2}]-1 \right\} \right.,$$

$$I_{4}^{(3,4)}=\cos^{2} \delta+\lambda_{\theta}\sin^{2} \delta.$$

The quantities $\lambda_{r}$,$\lambda_{\theta}$ and $\lambda_{z}$ are geometric stretch factors in the radial, azimuthal and axial directions respectively and $\beta$, $\gamma$ and $\delta$ are the angles between collagen fibers embedded in intima, media, and adventitia. These families of fibers [37] are oriented in a helical fashion within each layer, rendering it strain-hardening and anisotropic. The constants $b_{0}$, $b_{1}^{(2)}$, $b_{2}^{(2)}$, $b_{1}^{(3,4)}, b_{2}^{(3,4)}$, $c_{0}$, $c_{1}^{(2)}$, $c_{2}^{(2)}$, $c_{1}^{(3,4)}$, $c_{2}^{(3,4)}$, $d_{0}$, $d_{1}^{(2)}$, $d_{2}^{(2)}$, $d_{1}^{(3,4)}$ and $d_{2}^{(3,4)}$ are material constants that characterize the mechanical properties of the intima, media and adventitia, and can be found from axial testing of specimens. As we discuss below, some of these parameters change in time to allow the simulation of atherosclerosis or MS.

**Stiffening of layers and estimation of stiffening rates**

**Intima**

The intima is taken to slowly stiffen over the course of a patient’s lifetime through the relation

$$b_{0}=b_{0}^{*}+\lambda_{s} t,$$

where $\lambda_{s}$ is the stiffening rate, which is estimated from data on coronary arteries (see below) and *t* is measured in years. Akyildiz et al. [38] give the Young’s modulus of a moderately stiff intima to be 500 kPa, so we use 500/3 kPa as the initial value of the shear modulus $b_{0}$: see S1 Table B.

Holzapfel et al. [39] measured the value of ${\mu=b}_{0}/2$in coronary intimas from 13 patients who were diagnosed with atherosclerosis in either aorta, coronary, cerebral, or renal arteries. The values of $\mu$ ranged from 15.93 kPa to 53.95 kPa while the age of patients ranged from 54 to 80 years. The stiffening rate $\lambda_{s}$ is therefore estimated as

$$\lambda_{s}=2 \left( \frac{53.93 kPa-15.93 kPa}{80-54} \right)\approx3 \text{kPa} \text{per year.}$$

**Media**

The stiffness of the media increases over time for both non-MS and MS patients, but at different rates. In the context of our model, this amounts to allowing${c_{0}, c}_{1}^{\left( 2 \right)}$, $c_{2}^{\left( 2 \right)}, c_{1}^{\left( 3,4 \right)}$ and $c_{2}^{\left( 3,4 \right)}$ to change in time. Currently, one-time measurements of material parameters${c_{0}, c}_{1}^{\left( 2 \right)}$, $c_{2}^{\left( 2 \right)}, c_{1}^{\left( 3,4 \right)}$ and $c_{2}^{\left( 3,4 \right)}$ are usually performed *in-vitro* after removing the arteries in question from the patient. Although such measurements are insufficient to determine the time-course of these parameters, longitudinal studies on animals could provide more insight into the evolution of${c_{0}, c}_{1}^{\left( 2 \right)}$, $c_{2}^{\left( 2 \right)}, c_{1}^{\left( 3,4 \right)}$and $c_{2}^{\left( 3,4 \right)}$.

In the absence of data, we assume that the media stiffens linearly over time so that

$$\left( \begin{matrix} c_{0} \\ c_{1}^{(2)} \\ c_{2}^{(2)} \\ c_{1}^{(3,4)} \\ c_{2}^{(3,4)} \end{matrix} \right)=\left( \begin{matrix} c_{0}^{*} +\lambda_{0} t \\ c_{1}^{\left( 2 \right)*} +\lambda_{1}^{\left( 2 \right)} t \\ c_{2}^{\left( 2 \right)*}+\lambda_{2}^{(2)} t \\ c_{1}^{\left( 3,4 \right)*}+\lambda_{1}^{(3,4)} t \\ c_{2}^{\left( 3,4 \right)*}+\lambda_{2}^{(3,4)} t \end{matrix} \right),$$

where simulation time *t* is measured in years and $c_{0}^{*}$, $c_{1}^{\left( 2 \right)*}$, $c_{2}^{\left( 2 \right)*}, c_{1}^{\left( 3,4 \right)*}$ and $c_{2}^{\left( 3,4 \right)*}$ are given in S1 Table C. The model is calibrated so that at *t=0* (time of birth), the mechanical properties of the media and adventitia are identical.

We estimate MS stiffening rates $\lambda_{0},$ $\lambda_{1}^{(2)}, \lambda_{2}^{(2)}, \lambda_{2}^{(2)} \text{and }\lambda_{1}^{(3,4)}$, from atherosclerotic arteries in Kamenskiy et al. [21]*.* Specifically, the arteries of Patients 5 and 10 in this study appeared to present atherosclerosis (and probably MS) at different stages. The vessels of patient 5 were essentially normal while patient 10 presented severe coronary artery disease. These two patients were 5 years apart in age and we estimate the stiffening rates by

$$\lambda_{0}=\frac{c_{0}\left( Patient 10 \right)-c_{0}\left( Patient 5 \right)}{5 years}.$$

A similar formula was used to estimate $\lambda_{1}^{(2)}, \lambda_{2}^{(2)}, \lambda_{1}^{(3,4)} \text{and }\lambda_{2}^{(3,4)}$: see S1 Table E. Non-MS stiffening rates are assumed to be 10 times smaller than MS rates. The values $c_{0}, c_{1}^{\left( 2 \right)}, c_{2}^{(2)}, c_{1}^{\left( 3,4 \right)}\text{and} c_{2}^{\left( 3,4 \right)}$ in S1 Table E come from different arteries in each patient. While this is obviously not ideal, we are not aware of any longitudinal studies that report how mechanical parameters of arteries evolve in time. Furthermore, note that the data in S1 Table E is used simply to estimate a stiffening *rate* rather than actual stiffness parameters.

**Adventitia**

We assume that the adventitia is not affected by MS, so its mechanical parameters are static in time: see S1 Table D. MS and non-MS adventitia always have the same mechanical properties.

**Atherosclerosis growth model**

Development of atherosclerotic lesions of the intima is represented by allowing *g* in the growth tensor to increase in time. Data from Osika et al. [19] suggests that intima growth is approximately linear in time with rate of about 1 $\mu$m per year. For a reference intima with thickness 10 $\mu$m, a growth of 1 $\mu$m per year corresponds to $g\left( t \right)=1+\lambda_{g}t$, with $\lambda_{g}=0.1$. A summary of intimal growth and mechanical parameters is given in S1 Table F.

**Tables**

**S1 Table A. Dimensions of Femoral and Tibial cross sections in their reference state.** Media/Adventitia thicknesses for femoral and tibial arteries are taken from [18, 40], respectively*.*

| Symbol | Meaning | Femoral Artery | Tibial Artery |
| --- | --- | --- | --- |
| A | Lumen radius | 3.5 mm | 1.5 mm |
| B-A | Intima thickness | 0.01 μm | 0.01 μm |
| C-B | Media thickness | 0.74 mm | 0.25 mm |
| D-C | Adventitia thickness | 0.42 mm | 0.11 mm |

**S1 Table B. Mechanical Parameters for the intima (MS and non-MS).** Taken from Kamenskiy et al. [21]*,* Patient 6’s Tibial Artery (TA (L)).

| $b_{0}^{*}$ (kPa) | $b_{1}^{\left( 2 \right)}$ (kPa) | $b_{2}^{(2)}$ | $b_{1}^{(3,4)}$ (kPa) | $b_{2}^{(3,4)}$ | $\beta$ (^o^) |
| --- | --- | --- | --- | --- | --- |
| 167 | 82.69 | 4.15 | 33.53 | 10.15 | 46.99 |

**S1 Table C. Mechanical parameters for the media at time *t = 0*.** Taken from Kamenskiy et al. [21] Patient 5’s Tibial Artery (TA).

| $c_{0}^{*}$ (kPa) | $c_{1}^{\left( 2 \right)*}$ (kPa) | $c_{2}^{\left( 2 \right)*}$ | $c_{1}^{\left( 3,4 \right)*}$ (kPa) | $c_{2}^{\left( 3,4 \right)*}$ | $\gamma$ (^o^) |
| --- | --- | --- | --- | --- | --- |
| 73.63 | 167.21 | 29.11 | 39.99 | 32.68 | 48.93 |

**S1 Table D. Mechanical Parameters for the adventitia (MS and non-MS).** Taken from Kamenskiy et al. [21]*,* Patient 5’s Tibial Artery (TA).

| $d_{0}$ (kPa) | $d_{1}^{\left( 2 \right)}$ (kPa) | $d_{2}^{(2)}$ | $d_{1}^{(3,4)}$ (kPa) | $d_{2}^{(3,4)}$ | $\delta$ (^o^) |
| --- | --- | --- | --- | --- | --- |
| 73.63 | 167.21 | 29.11 | 39.99 | 32.68 | 48.93 |

**S1 Table E. Media mechanical parameters and estimated stiffening rates for MS patients.** Stiffening rates for non-MS patients are assumed 10x smaller. Data is from Patient 5 and Patient 10 from Kamenskiy et al. [21] who presented atherosclerotic disease at different stages. Stiffening rates were estimated by assuming a linear law for ${c_{0}\left( t \right), c}_{1}^{\left( 2 \right)}(t)$, $c_{2}^{\left( 2 \right)}(t), c_{1}^{\left( 3,4 \right)}(t)$ and $c_{2}^{\left( 3,4 \right)}(t)$; more details are given in the text.

|  | $c_{0}$ (kPa) | $c_{1}^{\left( 2 \right)}$ (kPa) | $c_{2}^{(2)}$ | $c_{1}^{(3,4)}$ (kPa) | $c_{2}^{(3,4)}$ |
| --- | --- | --- | --- | --- | --- |
| Patient 5 (55 years old) | 31.87 | 130.16 | 26.09 | 8.80 | 27.90 |
| Patient 10 (60 years old) | 35.60 | 158.56 | 47.45 | 13.87 | 30.22 |
| Estimated Stiffening Rate for MS arteries | $\lambda_{0}$ | $\lambda_{1}^{\left( 2 \right)}$ | $\lambda_{2}^{(2)}$ | $\lambda_{1}^{(3,4)}$ | $\lambda_{2}^{(3,4)}$ |
|  | 0.75 kPa/year | 5.7 kPa/year | 4.3/year | 1.0 kPa/year | 0.5/year |

**S1 Table F. Summary of intima mechanical and growth parameters.**

| **Symbol** | **Value** | **Meaning** |
| --- | --- | --- |
| $\lambda_{g}$ | 0.1/year | Intima Growth Rate |
| $b_{0}^{*}$ | 167 kPa | Initial Intima Stiffness |
| $\lambda_{s}$ | 3 kPa/year | Intima Stiffening Rate |

**References**

1. Guyton JR, Hartley CJ, Flow restriction of one carotid artery in juvenile rats inhibits growth of arterial diameter. *Amer. J. Physiol.-Heart and Circulatory Physiol*, **248**, H540–H546 (1985).
2. Akyildiz A, Speelman L, van Brummelen H, Gutierrez MA, Virmani R, van der Lugt A, et al., [*Biomed. Eng. Online*](https://www.ncbi.nlm.nih.gov/pubmed/?term=)+Effects+of+intima+stiffness+and+plaque+morphology+on+peak+cap+stress.+Biomedical+Engineering+Online+10(25)%3A)*,* Effects of intima stiffness and plaque morphology on peak cap stress. Apr 8;**10**:25. doi: 10.1186/1475-925X-10-25, (2011).
3. Holzapfel GA, Sommer, G, Gasser CT, Regitnig P Determination of layer-specific mechanical properties of human coronary arteries with nonatherosclerotic intimal thickening and related constitutive modeling. *Am. J. Heart Cir. Physiol.* **289**, H2048 – H2058 (2005).
4. Sarkola T, Jaeggi E, Slorach C, Hui W, Bradley T, Redington AN, Assessment of vascular remodeling after the Fontan procedure using a novel very high resolution ultrasound method: arterial wall thinning and venous thickening in late follow-up. *Heart Vessels* **28**, 66 – 75 (2013).
